# Effects of Integrated Traditional Chinese and Western Medicine for the Treatment of Lupus Nephritis: A Meta-Analysis of Randomized Trials

**DOI:** 10.1155/2016/1502107

**Published:** 2016-12-26

**Authors:** Mingli Heng, Jinli Tu, Yu Hao, Ye Zhao, Jinhui Tian, Huaien Bu, Hongwu Wang

**Affiliations:** ^1^Department of Public Health, Tianjin University of Traditional Chinese Medicine, Tianjin 300193, China; ^2^Department of Language and Culture, Tianjin University of Traditional Chinese Medicine, Tianjin 300193, China; ^3^Department of Library, Tianjin University of Traditional Chinese Medicine, Tianjin 300193, China; ^4^Department of Chemical Engineering, University of Florida, Gainesville, FL 32611, USA; ^5^Evidence-Based Medicine Center, Lanzhou University, Lanzhou 730000, China

## Abstract

After a thorough search through the database as CNKI database, VIP database, Wanfang database, PubMed, and Cochrane Library, the clinical experimental articles have been selected out on the effects of Integrated Traditional Chinese and Western Medicine on the treatment of lupus nephritis. A meta-analysis was carried out in terms of clinical efficacy criteria and safety criteria by RevMan 5.3 software. Based on the results, we cautiously conclude that Integrated Traditional Chinese and Western Medicine used for lupus nephritis could improve the clinical efficacy while at same time lower the 24-hour urine protein, serum creatinine, and adverse drug reactions.

## 1. Introduction

Lupus nephritis (LN) is a kind of kidney impairment caused by systemic lupus erythematosus (SLE) [1]. For 25% of SLE patients, renal involvement will be the primary manifestation. About 50% SLE patients will suffer from kidney impairment during the disease progress, which eventually will develop into lupus nephritis. Almost every SLE patient examined by renal biopsy will show the sign of kidney impairment more or less. As much as 30% cases of lupus nephritis will worsen into end-stage renal disease in 15 years after diagnosis [2–5].

Lupus nephritis is a common but severe systemic impairment caused by systemic lupus erythematosus. It is the fatal reason for SLE patients, which needs to be treated positively [6]. In modern medicine, glucocorticoid, immunosuppressor, and biologics have been adopted widely to delay the disease progress remarkably, but with significant effectiveness for only 70%–80% patients, and at same time with serious adverse reaction [7, 8].

Many studies showed that Integrated Traditional Chinese and Western Medicine (Integrated Medicine) played a positive part in treating lupus nephritis; however with small size of multisamples and different articles intermingled, it was rather difficult to reach a reliable conclusion just based on the small sample size of RCTs. This research was aimed at conducting a systematic review on the studies of the treatment of lupus nephritis by Integrated Medicine and at performing a meta-analysis on some vital criteria, with the intention of providing the evidence for treatment of lupus nephritis by Integrated Medicine in the viewpoint of Evidence-based Medicine.

## 2. Methods

### 2.1. Inclusive Criteria

#### 2.1.1. Study Pattern

The studies, published in China and elsewhere on the RCTs about the treatment of lupus nephritis by Integrated Medicines, were selected. According to Cochrane practices, there is no limit on the adoption of blind method in the research. The studies were published in the language of Chinese or English.

#### 2.1.2. Study Subject

The study subjects were the patients who were definitely diagnosed with lupus nephritis based on the American college of rheumatology revised criteria for the classification of systemic lupus erythematosus issued in 1997. The patients would be *⩾* 14 years old with no limits on their genders and case resources; however the women in pregnancy were excluded.

#### 2.1.3. Intervention

Traditional Chinese medicine (TCM) and Western Medicine (WM) were both applied in the treatment group as intervention, while Western Medicine alone was adopted in the comparison group. TCM was used in the form of decoction, pill, capsule, and patent medicine. The treatment would last for at least three months with sample size *⩾* 10 cases.

#### 2.1.4. Outcome

*Efficacy Criteria*. The primary efficacy criteria are clinical efficacy rate and the secondary efficacy criteria are 24-hour urine protein and serum creatinine.

*Safety Criteria*. The adverse drug reactions are adopted as the safety criteria. 

### 2.2. Exclusive Criteria

① Duplicated articles.
② Nonclinical trials such as animal test, pharmacology, and pharmacokinetics.
③ Non-RCT studies such as literature review, expert experiences, and mechanism elaboration.
④ Nonrandomized compared trials, compared trials with Chinese medicine in different dosage.

### 2.3. Articles Search

The search was conducted via computer across the following databases: CNKI database, VIP database, Wanfang database, Cochrane Library, and PubMed on the studies published in Chinese or English between January 1, 1997, and August 1, 2015. The search items were lupus nephritis, Integrated Medicine, Traditional Chinese Medicine, and Chinese herbal medicine.

### 2.4. Quality Assessment of Included Studies

The quality of the trials included in this study was assessed by each researcher according to the Cochrane collaboration's tool for bias risk assessment. The assessment was performed on ① randomized allocation method; ② allocation concealment; ③ blind method; ④ the completion of outcome statistics; ⑤ selective research outcomes; ⑥ other bias, such as the symmetry of base line, the cheating behavior, or any conflicts of interests in the research. The outcomes of above 6 items were evaluated as low risk, unclear, and high risk.

### 2.5. Data Extraction and Analysis

Two investigators independently extracted the data with same extraction chart regarding basic information, intervention, observation time, outcomes criteria, and research outcomes. The third researcher would be invited for discussion whenever different opinions appeared. The statistics analysis was performed with Review Manager 5.3 from Cochrane. Risk Ratio (RR) was used for count data while Mean Difference (MD) was adopted for continuous variables as effect size, respectively, both of which were demonstrated with effect size and 95% confidence intervals (CI). The chi-squared test and the *I*-squared statistic were used to assess the heterogeneity. Fixed-effect model would be adopted when *P* > 0.1 or *I*^2^ < 50%. Random-effect meta-analysis model would be used when *P* < 0.1 or *I*^2^ > 50%. Descriptive methods would be applied if the data was insufficient.

## 3. Results

### 3.1. Database Search

Initially 605 publications were identified, among which 273 articles are from CNKI database, 173 articles from VIP database, 159 articles from Wanfang database, zero article from PubMed from database, and zero article from Cochrane Library. Through careful selection, six studies [9–14] were included with 470 cases. The Western Medicine therapies used in six studies included the treatment of patient's primary disease in order to exclude the inducing factor and the use of glucocorticoid and cyclophosphamide.

### 3.2. Study Characteristics

It is shown in Table 1.

### 3.3. Quality of the Included Studies

It is shown in Table 2.

### 3.4. Meta-Analysis of Outcome Criteria

#### 3.4.1. Clinical Efficacy Rate

**Figure 1:**
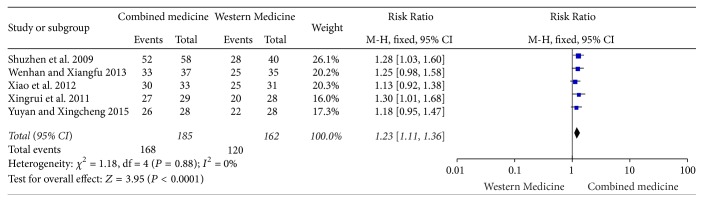
Effective rate of clinical efficacy rate between two groups.

Five studies [9–13] demonstrated the clinical efficacy of the Integrated Medicine used for lupus nephritis. There was no significant heterogeneity (*I*^2^ = 0%, *P* = 0.88). The meta-analysis was conducted on fixed-effect model. The outcomes indicated the clinical efficacy rate in Integrated Medicine group was higher than that in comparison group. There was significant difference [RR = 1.23, 95% CI (1.11, 1.36), *P* < 0.0001] (Figure 1).

#### 3.4.2. 24-Hour Urine Protein

**Figure 2:**
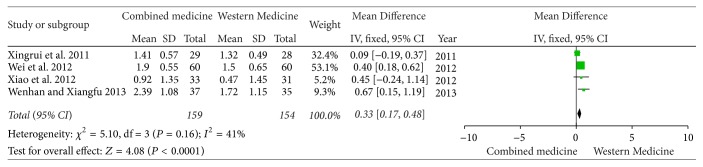
24-hour urine protein between two groups.

Four studies [10–12, 14] reported the changing of 24-hour urine protein before and after treatment. There was no significant heterogeneity (*I*^2^ = 41%, *P* = 0.16); therefore the meta-analysis on fixed-effect model was performed. There was significant difference in lowering 24-hour urine protein between two groups [MD = 0.33, 95% CI (0.17, 0.48), *P* < 0.00001] (Figure 2).

#### 3.4.3. Serum Creatinine

**Figure 3:**
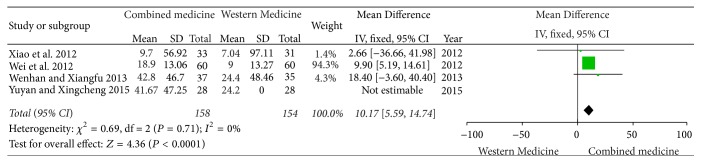
Serum creatinine between two groups.

Four studies [11–14] showed different serum creatinine before and after treatment in two groups. There was no significant heterogeneity (*I*^2^ = 0%, *P* = 0.71); therefore the meta-analysis on fixed-effect model was carried out. There was significant difference in lowering serum creatinine in two groups [MD = 10.17, 95% CI (5.59, 14.74)] (Figure 3).

#### 3.4.4. Adverse Drug Reaction

**Figure 4:**
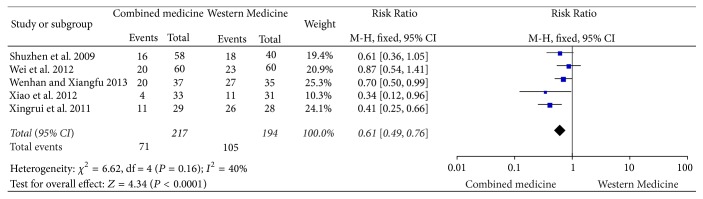
Adverse drug reaction between two groups.

Five studies [9–12, 14] indicated adverse drug reaction in the treatment. There was no significant heterogeneity (*I*^2^ = 40%, *P* = 0.16); therefore the meta-analysis on fixed-effect model was used. The outcomes indicated the adverse drug reaction in Integrated Medicine group was lower than in comparison group. There was significant difference [RR = 0.61, 95% CI (0.49, 0.76), *P* < 0.0001] (Figure 4).  Table 3 shows the nature of adverse drug reactions.

#### 3.4.5. Funnel Plot

**Figure 5:**
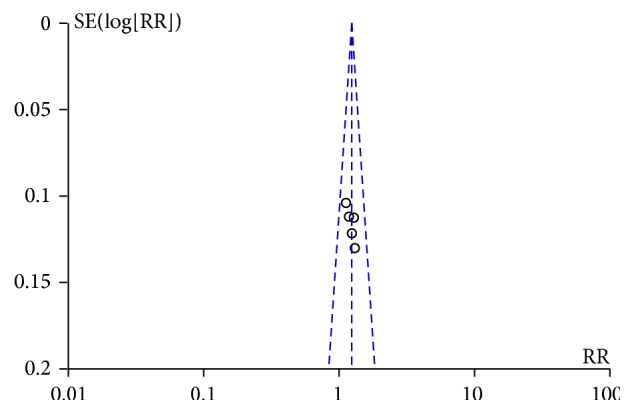
Funnel plot.

Clinical efficacy rate was adopted as the outcome criteria to compare Integrated Medicine with Western Medicine in treating lupus nephritis. Funnel plot analysis was conducted based on these five studies included. The results indicated that the funnel plot was in graphic symmetry. Owing to the small amount of articles included, the funnel plot results were not stable (Figure 5).

## 4. Discussion

### 4.1. Efficacy Analysis of Treatment of Lupus Nephritis by Integrated Medicine

Cyclophosphamide and glucocorticoid were the common therapies to treat lupus nephritis, which could control the disease progress, promote renal function effectively, and improve the survival rate. However, there would be some adverse effects, including amenorrhea and atrophy of ovary or with the impossibility of the increase of infection as well as herpes zoster and hemorrhagic cystitis [15]. The disadvantages to treat lupus nephritis with TCM were listed as follows: firstly, taking Chinese herbal medicine orally will cause gastrointestinal tract irritation and aggravate clinical symptoms. Secondly, the treatment would be slow in efficacy. The therapy of Integrated Medicine has been the main tendency in clinical research. The studies [16–19] indicated that the therapy of Integrated Medicine is better than the therapy of Western Medicine. During the active period, immunosuppressor was combined with Chinese herbal medicine to improve efficacy and relieve adverse effects, while, during remission period, Chinese herbal medicine would be helpful to make the case stable, decrease the Western Medicine used, and prevent the disease from reoccurrence. This paper searched out the articles based on the standards from* the American college of rheumatology revised criteria for the classification of systemic lupus erythematosus*, which would guarantee the advancement and timeliness of the research included. The results showed that, compared with routine Western Medicine treatment, the Integrated Medicine could not only further improve clinical efficacy and decrease 24-hour urine protein and serum creatinine but also lower adverse reaction because the Chinese herbal drugs used in this group have the function of eliminating heat and adverse effects, of nourishing liver and kidney, and of promoting blood circulation and removing stasis. This showed that Integrated Chinese is a comparatively safe and reliable intervention therapy to treat lupus nephritis.

### 4.2. Summary on Methodology Quality of Systematic Assessment on the Articles Included

The results showed that the quality of the methodology used in the six RCTs was on the lower level, which was manifested as follows: there was no or only a brief description about the randomization principle such as the generation of random sequence; there was no introduction about whether allocation concealment or blind method was adopted in the research or not; there was no mention on midterm withdrawal, loss of follow-up, number of excluded cases, and its reasons; and whether there was other bias or not was not clear. In terms of the feasibility and applicability of varied assessments used in clinical practice, the results gained by RCTs were generally regarded as the Gold Standard to prove the clinical evidence for its high quality and reliability [20]. However, these questions existing in methodology not only were disadvantageous for the readers to evaluate the research design, research implementation, data statistical analysis, and outcome reliability but also possibly lead to bias results, which would exaggerate the intervention efficacy and influence clinical decision. Hence, it was necessary to improve the methodology quality of the RCTs. At present, the systematic assessment has been conducted on quality of the methodology and reports of RCTs published in key Chinese nursing journals [21]. It was advisable for relevant researchers to design, implement, analyze, and compose RCTs researches on Integrated Medicine according to CONSORT criteria and Cochrane bias risk assessment tools, so as to improve the quality of the research report and provide advanced evidence for Evidence-based Medicine.

### 4.3. Limitation

The research indicated that it was obviously effective to treat lupus nephritis with Integrated Medicine, which was apparently superior to Western Medicine in total effective rate, lowering 24-hour urine protein and serum creatinine and decreasing adverse reaction. But the above-mentioned conclusion could not be spread due to the following reasons: ① quality of the included studies: because of the small amount of the RCTs included in systematic review, there were comparatively fewer total samples and lower methodology quality; ② research subject: lupus nephritis may be caused by various reasons such as genetic diseases, virus infection, dysimmunity, sunlight or ultraviolet radiation, and certain medicines [22] or with unclear reasons; since only small amount of studies was included in this review, which was unable to cover all lupus nephritis patients, the conclusion would show certain limitations; ③ intervention: for the six RCTs included in this review, there was no uniform TCM therapy used; this would influence the comparison between different experimental results and make it more difficult to explain the results; ④ conclusive criteria: the articles included in this review took total effective rate, 24-hour urine protein, serum creatinine, and adverse reaction as conclusive criteria, which were short of the follow-up of long-term drug effectiveness and the patient survival. Some research [7] indicated that treating lupus nephritis with Integrated Medicine had the advantage of stable function, good efficacy, lower price, and smaller adverse reaction. This could demonstrate the great advantage of TCM holistic treatment, which would increase white cell, improve clinical symptoms, and enjoy the future of wide application.

### 4.4. Inspiration for Future Research

After the systemic review of the six articles with 467 cases, the research found out that, compared with Western Medicine, treating lupus nephritis with Integrated Medicine was a comparative safe and effective intervention measure, which take obvious advantage to improve the total effectiveness and lower the adverse reaction. But this would not exclude the possibility that some experiments just reported the efficacy but ignored the adverse reaction. Therefore, the safety of Integrated Medicine needed to be further verified. In addition, it has been widely proved that the Integrated Medicine was effective to treat lupus nephritis, but its mechanism was not yet fully disclosed. So it was suggested that, in the future research, the relevant researchers should design more multicenter large-sample double-blind RCTs and establish uniform TCM diagnosis, treatment, and efficacy criteria, which was helpful to fully demonstrate the mechanism of treatment of lupus nephritis with Integrated Medicine and to further test the efficacy, mechanism, and safety of the treatment of lupus nephritis by Integrated Medicine.

## 5. Conclusion

Integrated Traditional Chinese and Western Medicine used for lupus nephritis could improve the clinical efficacy.

## Figures and Tables

**Table 1:** Study characteristics.

Study	Cases (male/female)		Duration (month)	Age (T/C, year)	Intervention		Outcomes measures
	Treatment group	Comparison group			Treatment group	Comparison group	
Shuzhen et al. (2009)	6/52	4/36	6	35.2/34.5	Xiaolang Jiushen Decoction + Western Medicine	Western Medicine	① ④
Xingrui et al. (2011)	2/28	3/27	6	14–47/17–48	Qingren Huoxue Huayu Decoction + Western Medicine	Western Medicine	① ② ④
Xiao et al. (2012)	1/32	1/30	5-6	41.1 ± 13.6/33.5 ± 13.4	Chinese herbal formulae + Western Medicine	Western Medicine	① ② ③ ④
Wei et al. (2012)	Not reported	Not reported	12	Not reported	Liuwei Dihuang Decoction Added with Tripterygium Glycosides + Western Medicine	Western Medicine	② ③ ④
Wenhan and Xiangfu (2013)	5/32	4/31	6	25.2 ± 9.5/24.9 ± 9.8	Liuwei Dihuang Pill Mixed with Qinghao Biejia Decoction + Western Medicine	Western Medicine	① ② ③ ④
Yuyan and Xingcheng (2015)	5/23	7/21	3	34.7±8.4/36.5±7.2	Shenqi Dihuang Decoction + Western Medicine	Western Medicine	① ③

CTX: cyclophosphamide; ①: efficacy rate; ②: 24-hour urine protein; ③: serum; ④: adverse drug reaction.

**Table 2:** Quality of the included studies.

Study	Random allocation method	Allocation concealment	Blind method	Completion of the outcome data	Selective report of outcome	Other bias resources
Shuzhen et al. (2009)	Unclear	Unclear	Unclear	100% completed	Unclear	No
Xingrui et al. (2011)	Unclear	Unclear	Unclear	100% completed	Unclear	No
Xiao et al. (2012)	Unclear	Unclear	Unclear	100% completed	No	No
Wei et al. (2012)	Unclear	Unclear	Unclear	100% completed	No	No
Wenhan and Xiangfu (2013)	Unclear	Unclear	Unclear	100% completed	No	No
Yuyan and Xingcheng (2015)	Unclear	Unclear	Unclear	100% completed	Unclear	No

**Table 3:** The nature of adverse drug reactions.

Study	Combined medicine	Western Medicine
Shuzhen et al. 2009	Side effect: alopecia Secondary response: concurrent infection	Side effect: alopecia Secondary response: concurrent infection

Wei et al. 2012	Side effect: fatigue, edema, nausea, poor appetite Secondary response: pulmonary infection, peptic ulcer Allergic reaction: increased AST and ALT	Side effect: fatigue, edema, nausea, poor appetite Secondary response: pulmonary infection, peptic ulcer Allergic reaction: increased AST and ALT

Xingrui et al. 2011	Side effect: subxiphoid discomfort Secondary response: moon-face, excessive hair growth, infection Allergic reaction: neurological symptoms, abnormal hemogram	Side effect: subxiphoid discomfort Secondary response: moon-face, excessive hair growth, infection, abnormal blood glucose Allergic reaction: neurological symptoms, abnormal hemogram

Wenhan and Xiangfu 2013	No detailed information	No detailed information

Xiao et al. 2012	Side effect: nausea, anorexia Allergic reaction: leucopenia	Side effect: anorexia, nausea, diarrhea Allergic reaction: pruritus, increased ALT, leucopenia
